# European Consumers’ Perceptions and Attitudes towards Non-Thermally Processed Fruit and Vegetable Products

**DOI:** 10.3390/foods9121732

**Published:** 2020-11-25

**Authors:** Xiao Song, Paola Pendenza, María Díaz Navarro, Elisa Valderrama García, Rossella Di Monaco, Davide Giacalone

**Affiliations:** 1 Department of Technology and Innovation, University of Southern Denmark, 5230 Odense, Denmark; xso@iti.sdu.dk; 2 Associazione Italiana Difesa Consumatori ed Ambiente (ADICONSUM), 00199 Rome, Italy; paola.pendenza@adiconsum.it; 3 Cluster FOOD+I, 26500 Calahorra La Rioja, Spain; mdiaz@clusterfoodmasi.es; 4 Ctic Cita, 26315 Alesón La Rioja, Spain; evalderrama@cticcita.es; 5 Department of Agricultural Sciences, Universita’ degli Studi di Napoli Federico II, 80055 Portici, Italy; rossella.dimonaco@unina.it

**Keywords:** non-thermal processing technologies, consumer perception, fruit and vegetables, food processing

## Abstract

In order to meet the demand for high-quality fruit and vegetable (F&V) products, a wide variety of novel non-thermal processing (NTP) technologies are under development. This study used a qualitative focus group approach to investigate consumers’ perception and attitudes towards non-thermally processed F&V products among young (18–30 years old) and middle-aged (45–60 y.o.) consumers across six European countries: Denmark, Germany, Italy, Serbia, Spain, and the Netherlands. Findings show that the expected benefits and social concerns are important factors which affect consumers’ attitudes toward non-thermally processed F&V. Extending shelf-life, being healthier and more nutritious, and better hygiene and safety were important benefits, whilst impacts on product quality, safety risks, higher price and environmental costs were the concerns most often mentioned by participants. However, due to a lack of knowledge and trustworthy information sources, consumers have difficulties in assessing relevant benefits and risks. Targeted communication that could explicitly and efficiently reveal benefits and risks is highly recommended to enhance consumer awareness and trust. This may especially be needed to reach middle-aged consumers who showed less confidence in NTP, compared to young consumers. Consumers from Spain, Denmark, and the Netherlands appeared more interested in consuming NTP F&V, compared to Italy, Serbia, and Germany. These findings are expected to provide recommendations to better communicate non-thermally processed F&V with consumers in the EU.

## 1. Introduction

Fruit and vegetables (F&V) are critical elements of a healthy diet supplying essential nutrients to humans [1]. The increase in consumers’ attention to the “healthy” food attributes (e.g., “freshness” and “naturalness”) and the overall sustainability of processing technologies has contributed to a growing demand for non-thermally processed F&V [2].

Food processing technologies are improved on a continuous basis. Currently, a wide variety of non-thermal processing technologies (NTP) are under development [3,4]. During the processing of F&V, NTP use mild temperatures and minimal amounts of physical and chemical processing aids. Compared to conventional thermal technologies which use invasive temperature or treatments (e.g., thermal pasteurization), NTP are expected to better preserve the original quality of food products and by-products, such as maintain the nutritional value, freshness and some other sensory attributes of F&V products for a longer time, and reduce the use of added preservatives [5,6]. Moreover, the extension of shelf-life could potentially help with reducing food waste for both households and producers [7,8,9].

While scientists may applaud the progress of food technologies, consumers are known to have more conservative attitudes towards food processing [10]. Consumer choices are influenced not only by the intrinsic features of the product, but also by the production characteristics, including the way the products have been processed [10]. For some particular processing methods, some consumers have developed preferences or dislikes (e.g., irradiation) based on their vague understanding of these technologies [11,12,13].

A number of factors are known to influence consumers’ perceptions of food technologies. For instance, consumers’ perceived benefits and risks were found to affect consumers’ attitudes towards new food technologies [6,14,15]. Consumers paid special attention to the effects of processing on food quality, safety, price, and naturalness [6,16]. Due to their lack of knowledge and familiarity with food technologies, consumers have been reported to rely on simple heuristics, such as the affect heuristic, natural-is-better heuristic, and trust heuristic, when evaluating them [3]. Moreover, individual-related factors, such as food technology neophobia level [14], sense of disgust evoked by the unfamiliar, and cultural values, further influence the acceptance of a technology [3]. These factors could lead to limited confidence and lack of acceptance in non-thermally processed products and difficulties in associating NTP with possible benefits or risks, especially when the benefits and risks were hard to be directly experienced by consumers [3,10,14].

Nevertheless, from a consumer policy standpoint, consumer-oriented communication is important to enhance social awareness and trust in products processed with novel technologies [16]. Interestingly, sufficient communication of the processing information has found to positively influence consumers’ perception of novel processing methods, especially when expressed by independent scientists, consumer organizations, or food safety authorities [6,14,17,18].

In light of the growing interest in mild processing for shelf-life extension and food waste reduction, this study aims to better understand consumers’ perception and attitudes towards non-thermally processed F&V, drawing on data from six European countries: Denmark, Germany, Italy, Serbia, Spain, and the Netherlands. Moreover, consumers’ perceptions towards the potential effects of interactive NTP on household F&V waste reduction were also investigated. Findings from this study are expected to provide recommendations to better communicate non-thermally processed F&V with consumers in the EU.

## 2. Materials and Methods

### 2.1. Participants

In total, 12 focus groups were conducted in six European countries with 94 participants. In order to obtain a pan-European outlook of consumers’ perception and attitudes, Denmark, Germany, Italy, Serbia, Spain, and the Netherlands were chosen for this research. In each country, two age groups were addressed: young-age (YA, 18–30 years old) and middle-aged (MA, 45–60 years old) population. These two groups were selected as target groups due to their known differences in purchasing power and attitudes towards novel food products and technologies [19,20]. Besides age and nationality, participants were screened by using the following criteria: (a) being responsible for grocery shopping; (b) not affiliated with the project; (c) not working professional with food and nutrition. Finally, each focus group had a balanced number of female and male participants. Table 1 shows the participants’ basic demographic information on a country by country basis.

### 2.2. Procedures

All the focus groups (FGs) were conducted between November 2019 and February 2020. The same protocol was followed throughout the discussion to ensure consistency across all six countries (Table 2). FGs were conducted in the native languages of the participating countries. Each group discussion lasted about 90 to 120 min. Both video and audio recordings were collected for the subsequent data analysis.

Before the FG discussion, participants received and signed an informed consent form describing the aim of the project and the use of the data, as well as a short questionnaire designed to collect their basic demographic information. The moderator started the group discussion with a brief introduction to the overall project and the procedure of the ensuing discussion. Then, the participants introduced themselves. Afterwards, the moderator followed the group interview protocol (Table 2) to discuss the themes one by one.

Participants’ perceptions towards NTP (theme 2) were discussed in three stages. Firstly, the moderator asked participants if they knew any F&V processing technologies and whether they’ve heard about NTP before. Then the moderator introduced the NTP concept briefly as follows: “*NTP use mild temperatures and limited amounts of physical processing aids to increase shelf-life and keep the nutrients, freshness and sensory attributes of F&V products for longer time.*” Afterwards, participants’ perceptions and attitudes towards NTP were further collected.

In the second stage, more detailed explanations of some representative NTP were presented to consumers, supported by PowerPoint slides. The moderator summarized the NTP into categories of sanitization, preservation, stabilization, and extraction and gave examples for each category: ultrasounds, electrolyzed water, plasma-activated water, blue light, and UV light for sanitization; bioactive coating, active and intelligent packaging for preservation; ultrasounds and high-pressure processing for stabilization; ultrasounds, pulsed electric field, and membrane filtration for extraction of bioactive compounds in F&V. In-depth discussions about participants’ perceptions towards NTP were followed, focusing on expected benefits and concerns regarding the NTP. The underlying reasons which caused the above concerns and expectations were explored at the end of stage 2.

The last stage focused on participants’ willingness to purchase non-thermally processed F&V and their expected communication approaches of the processing information provided by different sources.

### 2.3. Data Analysis

Recordings of the FG discussion from the six countries were translated and transcribed into English text. NVivo 12 (QSR International, Warrington, UK) was used to code the transcriptions based on the standard content analysis procedures [21,22,23,24]. Various codes were compared and sorted into factors and categories based on similarities and differences, addressing the corresponding themes. Results are presented with a focus on the most recurrent stated factors and discussed both in general as well as based on the participants’ demographic background. Representative quotes of participants from different countries are included to further support and illustrate the relevant claims and findings.

## 3. Results

In accordance with the focus group themes (Table 2) the results are sequentially presented in the following order: (1) participants’ preferred quality attributes of F&V; (2) participants’ knowledge and perceptions toward NTP; (3) participants’ use of product information at the point of purchase; (4) household storage and potential effects of interactive NTP on the waste of F&V.

### 3.1. Preferred Features of Fruit and Vegetables at Point of Purchase

**Figure 1:**
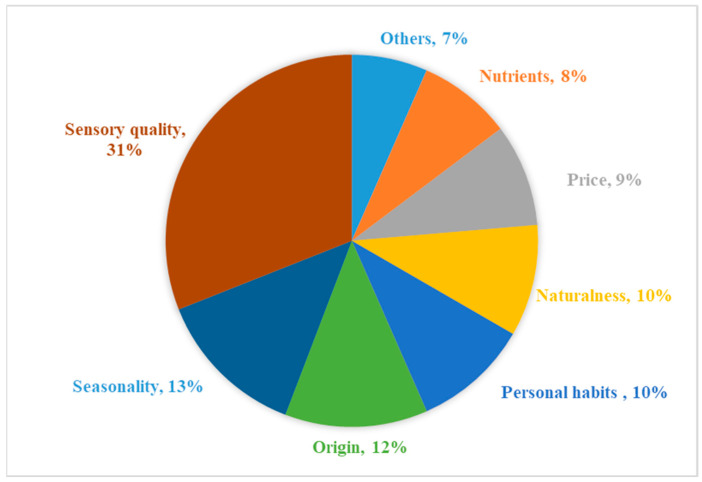
Frequency distribution of factors considered at point of purchase.

In Figure 1, the pie chart illustrates the frequency distribution of the factors that influenced the purchase of F&V products, based on the responses from all 94 participants collected at the discussion of theme 1.

The factors can be summarized into three major categories: (1) internal product features, such as sensory quality (31%), seasonality (13%), origin (12%), naturalness (10%) and nutritional value (8%); (2) external product features; and (3) personal habits and individual needs of consumers.

With regards to sensory quality, participants paid most attention to product appearance and taste. Accordingly, product appearance was mentioned as the first cue for freshness for participants. Moreover, participants reported that they sometimes tried to smell and touch the products to tell their freshness. Local products or products originating from areas with a good reputation and in the right season(s) were preferred. Participants generally thought local and seasonal products had a more natural taste and were more environmental-friendly. Many participants expressed a specific preference towards organic products and concerns over whether the products contain additives and preservatives. Compared to fresh produce, some participants tried to avoid purchasing F&V derived juices and smoothies due to the sugar and additive content in some industrial products, and instead preferred to make their own juices and smoothies. Some participants from Denmark mentioned a specific preference for products with the green-keyhole label [25], which is a Nordic certification for healthy foods.

There were some interesting discussions about the correlation between high quality and a beautiful appearance. While some participants believed that a good appearance is an important indicator of freshness, others declared that they were less willing to buy products which look “too perfect”, because they believed that too beautiful products were less natural and therefore may not taste good, as illustrated by the following quotes:

*“The inconsistency of apple size is more appealing to me. When I went to Korea, every apple was exactly the same size and that is too perfect.”*

(23 years old, male, Danish)

*“Things that are too beautiful always hide some imperfections.”*

(52 years old, female, Italian)

Price (9%) was the most frequently mentioned external product feature, followed by others including brand, package type (e.g., packaging materials), and the Fairtrade label (others, 7%). Participants from Denmark and Germany seemed more willing to purchase products with fair-trade certifications.

Because of differences in income and individual concerns, price was used differently as a quality cue by consumers. Some perceived products offered at particularly low prices to be of poor quality, whereas other participants preferred lower-priced products if there are no discernible differences in quality, compared to the more expensive ones, as the following quotes illustrate:

*“The low-priced juices are full of preservatives.”*

(26 years old, male, Italian)

*“A medium quality product must have an adequate price because no one gives you anything good for free. If there is a good relationship between price, quality, origin and authenticity, even if it is not branded, the product is acceptable for me.”*

(52 years old, female, Italian).

*“I will take the cheaper one first, unless the other one has something recognizable at first glance, it has the organic label on it, or something like this. Otherwise I don’t have the patience to compare.”*

(22 years old, female, German)

*“As a student, I just prefer products that are cheaper.”*

(23 years old, male, Dutch)

Participants’ personal habits (10%) played an important role in their F&V choices as well. Some participants preferred more convenient products, e.g., vegetables in cans, due to lack of time or interest in cooking, and paid less attention to nutrients damage or taste. Moreover, individual shopping frequency and plans determined choices of the package size and ripeness status of F&V, etc.:

*“As for vegetables, we consume a lot of canned ones. It is more convenient than cooking the vegetables every day. You go down to the supermarket and buy several jars, and you can preserve them better.”*

(27 years old, male, Spanish)

### 3.2. Knowledge and Perceptions toward Non-Thermal Processing Technologies

The discussion of theme 2 was conducted in three stages (Section 2.2). The first two stages were focused on participants’ knowledge and perceptions towards NTP.

#### 3.2.1. Initial Knowledge and Perceptions towards NTP

At the first discussion stage, participants reported a lack of knowledge regarding the processing of F&V products in general. Most of them showed concern about what happens during cultivation, with frequent mention of pesticides. With respect to the post-harvest processing, participants thought that some F&V products were processed in order to extend the shelf-life and enhance the quality, but save for a few exceptions (e.g., a few participants mentioned that they have heard that some F&V are coated with wax or sprayed with preservatives) participants did not have much specific knowledge. With respect to the concept of non-thermal processing technologies (NTP), almost all participants had never heard of it, with both quotes and their facial expressions confirming a complete lack of knowledge.

#### 3.2.2. Participants’ Concerns and Expected Benefits of NTP

At the second stage, after being introduced by the moderators to some of the most representative NTP, a few of the participants declared that they were familiar with the concepts of some technologies, for instance, blue light and ultrasound, but not in the context of food processing.

Moreover, participants were found to have various perceptions towards different NTP types after the introduction of representative NTP technologies. Some participants found some of the NTP techniques were relatively easier to understand and more acceptable, for instance, light-based technologies and active packaging.

*“Washing is the least bad … coating is the worst for me, you literally put it in your mouth so I wonder how good it could be.”*

(21 years old, female, Dutch)

**Figure 2:**
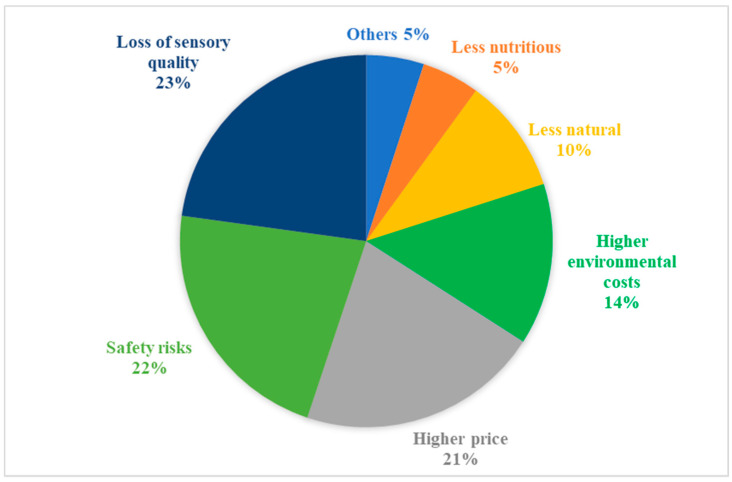
Frequency distribution of participants’ concerns towards non-thermal processing technologies (NTP) and NTP processed fruits and vegetables (F&V).

Some participants felt hesitant, or even outright objected, to purchase non-thermally processed F&V. The pie chart in Figure 2 illustrates the frequency distribution of the concerns of non-thermally processed products, based on the responses from all 94 participants at the second discussion stage.

Participants’ concerns regarding damages to the sensory quality of products were most frequently stated (23%). Accordingly, they stated that they would be more open to consuming non-thermally processed products if compared to conventional processing technologies, NTP did not cause loss of taste and aroma while maintaining the nutritious value and naturalness as close to the original (non-processed) products as possible:

*“The taste is very important. I have lived in South America and the bananas there are tastier. While they have to come all the way from there to our supermarkets, you need this kind of technology.”*

(23 years old, male, Dutch)

*“Everyone’s producing them now not to have taste and smell, but to look pretty. I don’t want to look at it, I want to eat it.”*

(46 years old, male, Serbian)

Regarding safety and health-related concerns, even though some of the participants acknowledged that compared to conventional technologies, NTP could reduce the use of chemical additives and preservatives in the final products, they were worried that it may introduce other harmful compounds into the products:

*“I think it’s important to say that these procedures assure me that I won’t get any kind of disease or any kind of bacteria by ingesting them.”*

(27 years old, male, Spanish)

*“How can that affect my health?”*

(26 years old, female, Serbian)

*“…look not only at profit but also at the good of the consumer, if you do not think that the product is intended for use by consumers, it could be dangerous to health.”*

(52 years old, male, Italian)

Moreover, some participants stated that if non-thermally processed F&V products were much more expensive than conventional processed or non-processed products, they would be less interested in them:

*“If it cleans 99% of the bacteria instead of 90%, then I don’t know if it’s worth for me to pay 10 or 20 cents more.”*

(23 years old, male, German)

Participants were concerned about energy costs and environmental impacts of NTP, for instance, whether the amount of water needed in the mild washing techniques would be higher than that of conventional washing. Interestingly, quite a few participants declared that they prefer to buy loose F&V than products with plastic packaging to reduce the environmental pollution that plastics can create, as exemplified by the following quotes:

*“The recyclability of the packaging matters as well.”*

(25 years old, female, Danish)

*“Maybe (novel) water washing leads to the use of larger amount of water, but with the use of lights you can use less water and therefore improve the fight against waste from this point of view.”*

(26 years old, male, Italian)

Moreover, a few participants associated extended shelf-life with loss of naturalness and nutrients and wondered whether too many chemical preservatives or too much treatment had been applied:

*“It’s not normal that something that should last for 2–3 weeks actually lasts 2 months.”*

(46 years old, female, Serbian)

*“…with the aim of preserving its freshness to make it more durable over time, but compromising its nutritional characteristics and taste is not natural.”*

(23 years old, female, Italian)

**Figure 3:**
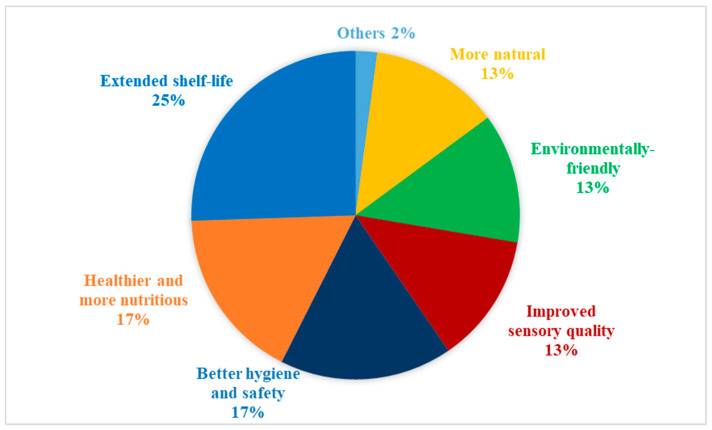
Frequency distribution of participants’ expected benefits of NTP and NTP processed F&V.

However, as illustrated in Figure 3, the extension of shelf-life in a “less invasive” way (25%) was one of the most important benefits expected by participants, compared to conventional processing. Moreover, they expressed positive expectations for the potential effect of NTP in the reduction of food waste due to the extended shelf-life, which could possibly enhance their interest in non-thermally processed products:

*“When it comes to shelf-life, we may have less food wasted, I think it’s an important item.”*

(55 years old, male, Dutch)

*“There are definitely benefits with the processing technologies. You do it for a reason. You do it to get rid of bacteria and germs to extend the shelf-life… you throw out less food and be able to ship it further over longer distance.”*

(25 years old, male, Danish)

Participants expected non-thermally processed F&Vs could be healthier and more nutritious (17%) than conventionally processed or non-processed products, due to the reduction of chemical preservatives and better maintenance of natural nutrients. Moreover, it was expected that non-thermally processed F&V could be more hygienic and therefore be safer to consume (17%), compared to non-processed F&V:

*“Perhaps the preserving of nutrients.”*

(25 years old, male, German)

*“When I hear the word ‘mild’, I’m assuming it’ll use much less chemicals … and other additives.”*

(58 years old, male, German)

*“These technologies give me an idea of disinfected, clean food, probably without microbes.”*

(56 years old, male, Italian)

Some participants expected that NTP could be better in the protection of products’ sensory quality (13%) and be more environmental-friendly (13%), comparing with conventional processing. A few participants regarded NTP as less invasive and more natural technologies in general:

*“If they are able to enhance its taste and texture and everything, then that is exciting.”*

(52 years old, female, Danish)

*“We even buy those products that are “heavily processed” already, as opposed, if it is processed by some mild version, I would probably prefer it.”*

(30 years old, female, Serbian)

At the end of stage 2, after thorough discussions of the factors which influenced participants’ perception of NTP and non-thermally processed F&V, the underlying reasons which caused the above concerns and expectations were explored. Some participants declared that their lack of knowledge and awareness due to the lack of complete and trustworthy information sources made them feel less confident in NTP and non-thermally processed products:

*“I am confused about what I don’t know.”*

(26 years old, female, Danish)

*“I don’t feel I have the knowledge to choose. I don’t know how to.”*

(52 years old, female, Danish)

*“Knowing about technologies, a person is more confident about what to buy.”*

(22 years old, male, Italian)

#### 3.2.3. Individual and Regional Differences

Participants could roughly be divided into four groups based on their various perceptions and interests towards NTP stated at the second stage of theme 2. The “accepting” group consisted of participants who were interested in NTP and willing to purchase non-thermally processed F&V with some prerequisites, such as that they caused no changes to the product property or added no chemicals into the products. They regarded NTP as a sign of scientific and technical progress that could improve F&V quality and reduce food waste in general:

*“It is ok as it is more natural and has no addition of the chemicals and stuff, like the washing and lights.”*

(25 years old, male, Danish)

*“I just think the science is very cool.”*

(25 years old, female, Danish)

The “neutral” group had limited interest in knowing NTP but were willing to purchase the treated products as long as the processing technology had been thoroughly tested and the quality of products was good and certified by trustworthy sources:

*“I honestly don’t mind processing. Just give me good, nice tasting apples, even if they’ve been really processed.”*

(23 years old, male, Danish)

*“It depends on how it really affects the product, if it is just to preserve it or if it can change some properties of the products, etc.”*

(54, female, Spanish)

The “rejecting” group had neither interest nor trust in NTP and were not willing to purchase F&V treated by NTP or any kind of processing technology. They regarded NTP as a marketing ploy and preferred non-processed, natural products.

*“The more a product is closer to the original status, without any processing, the better.”*

(22 years old, male, Serbian)

Finally, there were some participants who belonged to the “mixed feeling” group since they raised both concerns and expected benefits towards NTP.

Additionally, regional differences within the EU, as well as differences between demographics, were identified (Table 3 and Table 4). Overall, young participants appeared less worried and showed more interest in NTP processed F&V products, compared to middle-aged participants. “Healthier and more nutritious” was the most important benefit expected by middle-aged participants (23%, data not shown). They felt more worried about the loss in sensory quality (28%) and nutrients (9%), compared to the young participants (17% and 2%, respectively). By contrast, “extension of shelf-life” attracted young participants the most (32%), whilst they had higher concerns towards the increased price (25%) and safety risks (25%), compared to the middle-aged participants (17% and 20%, respectively). The middle-aged participants in four countries (Spain, Denmark, Netherlands, and Serbia) had more concerns towards the application of NTP, compared to the middle-aged participants in Italy and Germany.

Some gender differences were found in individual countries and specific benefits and concerns. Females from Spain and Serbia expressed fewer concerns towards NTP, whilst females from Denmark, the Netherlands, and Italy had more concerns, compared to males. Female participants were more interested in “better hygiene and safety” (22%) and “more natural” (17%), compared to male participants (13% and 8%, respectively). Male participants expected “extension of shelf-life” (33%) the most and showed more concerns to the price (24%) and loss in sensory aspects (24%), compared to female participants (17%, 19%, and 20%, respectively).

**Figure 4:**
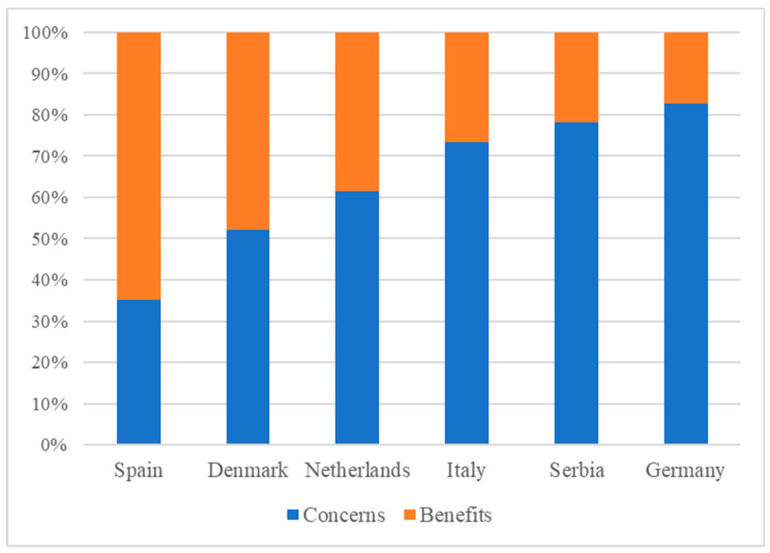
Percentages of concerns and expected benefits stated by participants in different countries.

With respect to differences across countries (Figure 4), participants from Spain and Denmark expressed fewer concerns and more expected benefits towards NTP, and appeared more open to learning how it works, compared to Serbia and Germany:

*“Suspicious. What is the actual process like? Will that affect my health and how?”*

(22 years old, male, Serbian)

*“I think they are mostly focused on having it look pretty.”*

(28 years old, female, Serbian)

*“I just think the science is very cool. It was washed with this water technology and then we put it through some blue light. Then we have smart vegetables … wow, this is science!”*

(25 years old, female, Danish)

*“I don’t know if it [i.e., the processing method, A/N] is important knowledge for me and I think I would rather trust the government. Living in Denmark, if the producers are allowed to produce and sell it, I would trust that I can just buy it without any risk.”*

(57 years old, male, Danish)

#### 3.2.4. Consumer Communication Aspects

At the third stage of theme 2, participants’ expectations towards communication of the processing information were discussed. Most statements focused on the type of language, the sources of information, the focuses of information, the communication channels, and the need for labels showing endorsement or certification.

Some participants hoped to better understand NTP by themselves and expect that packaging and social media could explicitly reveal the information in an efficient way, using language that average consumers can easily understand. On the contrary, other participants would rather let the experts and researchers decide what is the good and safe processing method and trust the products with relevant certifications in the market. They expected that the information sources could be trustworthy, monitored by relevant authorities, confirmed by experts, and in compliance with rules and regulations. Furthermore, many participants consequently stated that if the effects of NTP on both the products and human health have been thoroughly studied and NTP have been widely used in the F&V industry, they would feel more confident in consuming non-thermally processed F&V in their daily life:

*“I would be 90% sure if it has a certification.”*

(26 years old, female, Italian)

*“I would definitely not trust it if the benefits information was from the producer’s side. They have an interest in selling more apples. So it would need to come from, for instance, an external source for it to be credible enough.”*

(25 years old, male, Danish)

*“I am not an expert. I can believe that mild processing is useful, but there must be a predisposed institute to confirm that it is a positive process.”*

(26, male, Italian)

*“If it is a widely used thing, then I would feel safer.”*

(52, female, Italian)

Some participants declared they lacked time or interest to go in-depth by themselves, thus, they expected that product labels could just include a certification logo or a few words to directly highlight the benefits and advantages of non-thermally processed products compared to conventional or non-processed products. Participants also suggested that a QR code could be put on the label so that consumers who want to learn more about the processing details could just scan and explore by themselves. Some participants mentioned that except for using labels, signs, and packages to convey the information, short videos shown on in-store displays could also be an optional channel to explain NTP to consumers. Furthermore, a few participants mentioned that supermarkets could have tasting corners for consumers to taste non-thermally processed F&V, with staff to introduce relevant information to consumers, which they believed could help consumers both understand NTP and be less skeptical if the taste is good:

*“If put the information on a package or a product, I would not read it … maybe put it somewhere else, a website or a QR code. If someone really wants to know and wants to go deeper, they can have that information.”*

(27 years old, male, Spanish)

*“I wouldn’t give it too much thought on how it was processed.”*

(57 years old, male, Danish)

### 3.3. Use of Packaging Information at Point of Purchase

In general, for fresh fruit and vegetable produces, participants paid attention to the information of product origin, date of packaging, recyclability of the package, and the presence of relevant certifications, e.g., organic labeling. Regarding F&V-derived juices and smoothies, participants gave priority to the ingredients of the products, especially the presence of additives and the sugar content, followed by the nutrient table, dates of packaging and expiration, storage instructions, recyclability of the package, and the presence of certifications. Participants who were allergic to some food compounds usually checked the allergens information.

In addition to the conventional information on the package, some participants mentioned that they would like to have information sources on the entire product value chain, from its production until it reached consumers’ hands. Some participants showed interest in knowing a detailed description of the origin of the product, and the duration from its production until it was put on the shelf. Moreover, participants who were especially interested in sustainability issues would like to know the carbon footprint of the product during the planting and processing, compared to the average carbon footprint of similar products. Most participants expected easy access to such kind of information through scanning QR codes, e.g.:

*“I looked at the organic jam and one of them had a small QR code on it, which you can scan easily. You’ll see a map there. My jam came from the farmer Müller and therefore it costs 2–3 euros more. And I was so interested and decided to buy it immediately … I have my mobile phone in my hand anyway … I’m more involved and I found the website was very clear and well presented, much easier than looking on the long labels on the back of the package.”*

(22 years old, female, German)

### 3.4. Household Storage and Waste Reduction

Generally, participants did not have any formal knowledge regarding storage; rather, most stated that they followed the storage ways of their parents or simply replicate the same storage conditions as the supermarkets or grocery shops from where they bought the products. For fresh products, only a few participants declared that they used to check the storage instruction from the label or searched online to confirm whether they chose the best storage methods. Accordingly, the participants’ behavior in terms of F&V storing was not always compliant with recommendations: for instance, some participants stored potatoes and onions inside the fridge.

Participants declared that only a limited quantity of F&V was thrown away, in general. Participants discarded different kinds of F&V, mainly due to the sensory decays of the F&V. Sometimes, they made mistakes in shopping plans and bought more amount than needed, or just forgot what they have at home beforehand. Some participants, most notably YA who lived alone, complained about the package size of some F&V being too big for their consumption needs, but they had no choices of smaller packages or loose ones.

Many participants reported increased awareness of the societal costs of F&V waste and had made some active efforts to reduce it, for example by making better shopping plans. Some used the extra amount to bake cakes, make jams and soups, or simply freeze some kinds of F&V to make smoothies or for other use in the future. Some participants mentioned that they used some apps or websites to make recipes for their leftover F&V at home.

*“I think the perception or the knowledge and consciousness about food waste has increased. If I see myself ten years ago or just five years ago, I threw out foods without thinking … But now with this focus on food waste, I feel much more guilty when I throw out foods.”*

(55 years old, female, Danish)

Participants had suggestions for the industry as well. Better possibilities for purchasing loose products and/or smaller packages were expected. Supermarkets could lower the price of less fresh F&V. Producers could also suggest some recipes for their F&V in different ripeness status, by printing in their package or hiding in some QR codes.

Interactive packaging with freshness indicator attracted interest from some participants, especially for the packaging of products whose ripeness is hard to tell by touching and/or looking. They saw the advantages from the convenience, food sanitation, and waste reduction point of view, e.g.,

*“Melon, pineapple, avocado … in products that are more complicated to know if they are ripe or not at first sight. If you have to touch them, I think it’s better to put the indicator.”*

(27 years old, male, Spanish)

## 4. Discussion

To meet consumers’ demand for safe F&V products of high quality, a wide variety of non-thermal processing technologies are under development [3,4]. In this study, focus groups were used to explore factors that influenced consumers’ perceptions of non-thermally processed F&V products, comparing across six European countries: Denmark, Italy, Germany, Serbia, Spain, and the Netherlands. Moreover, in order to develop guidelines for the successful introduction and consumer communication of non-thermally processed F&V products, additional topics were discussed, including consumers’ use of and expected food processing technology information and other packaging information at the point of purchase and during household storage. Our findings showed that from the consumers’ point of view, even objectively less hazardous processes like non-thermal, mild processing could engender concerns which, if unattended, may override the benefits that the technologies could bring.

Lack of knowledge among the participants was one of the major impediments to their acceptance. Participants were found to largely rely on affect heuristic and trust heuristic when building their perceptions towards NTP [3]. When asked to evaluate the risks or benefits of NTP, participants associated with unknown food processing technologies with various food hazards, which evoked feelings of dread and influenced their benefit perceptions or risk judgments. The difficulties in assessing relevant benefits and risks could further impede the establishment of social trust and pose a barrier to the market introduction of non-thermally processed F&V [26]. One way that participants coped with their lack of knowledge is to rely on their trust in familiar brands or certified labels to reduce the complexity of making choices [18]. Regarding the individual-factors, disgust sensitivity and food culture and safety values were found to further influence the acceptance of NTP and explained the individual differences among participants [3]. Furthermore, consumers are grocery shopping with an ever-expanding perspective on overall health and well-being [19,25]. In addition to health, consumers’ desires for taste, food safety, affordability, convenience, and clear labeling and transparency were identified [6,14,17], which explained the major expected benefits and concerns stated in our focus groups. The increasing environmental awareness and sustainable thinking among consumers help them associate NTP with environmental cost [10,27], which was found to be one of the important factors that influenced consumers’ perceptions towards non-thermally processed F&V.

Consumer-oriented sufficient communication and early involvement of target consumers could contribute to a higher level of social trust in NTP and the likelihood of market success [4].

### 4.1. Recommendations on Development of Successful Consumer Communication

To counteract the effect of concerns about NTP on consumers’ food choice, consumer communication and education from technology development till product launch are highly recommended [16].

Our results revealed many factors that may affect the success of consumer communication with regard to NTP and influences caused by NTP, including trust in the information source, content and focus of the information, message development in terms of language and style, and communication channels.

- *Information source*

If consumers do not trust the information source, the benefits may not be convincing. Consumers expect that information sources should be trustworthy, monitored by relevant authorities, confirmed by experts from third parties, and in compliance with rules and regulations. This finding fits with research by Siegrist [14], who reported that when benefits are endorsed by independent organizations or scientists, the communication is more likely to positively influence consumers’ interests in consuming food products processed by novel technologies.

- *Information content*

Participants showed that they were hesitant to accept novel NTP mainly because they were not aware of any potential safety or health risks and clear benefits, due to lack of information. They expected that the public could be informed about the proven safety and benefits of NTP in a sufficient way, based on which they could be more likely to accept a novel food technology. This finding is in line with previous research reporting that tangible benefits based on consumer needs and expectations could reduce misunderstanding and positively affect consumers’ perceptions and purchase willingness [4,28].

For the development of communication messages, it is recommended to focus on the most important perceived benefits of NTP included enhanced quality and safety, extension of shelf-life, lower environmental impacts, and maintenance of naturalness and nutrients. These findings are also in line with previous studies [10,27,29,30,31] and could be emphasized in consumer communication and marketing campaigns as appropriate. It should be noted that compared to benefits for consumers, industry benefits may not have a significant effect on consumers’ purchase intention [14].

The extension of shelf-life is one of the major goals as well as benefits of NTP, which could potentially contribute to the reduction of F&V waste. Even though this benefit was expected by most participants in this study, some participants associated longer shelf-life with the addition of preservatives and loss of taste and naturalness. This finding should be of concern to marketers regarding the communication of benefits related to shelf-life.

- *Language style*

Companies should not assume that consumers may view specific technical terms the same way as they do [26]. In the past, consumers were regarded as passive receivers of product information. Yet, consumers were often found to misunderstand or misinterpret the information [4,14,27].

With regards to communication of non-thermally processed products, it should be of concern to marketers of non-thermally processed F&V that the phrase “non-thermal processing technologies” and “minimal processing” may have negative utility for some consumers. Indeed, NTP may only be regarded as a benefit by food technologists and nutritionists who are aware of the negative effects of thermal processing on food sensory quality and nutritional value [16,26]. By contrast, our findings indicate that for some consumers, NTP may be received as a marketing trick to increase the product price. Moreover, some consumers rejected F&V processed by any method because they saw them as less natural and would rather purchase F&V without any preservation technology. Thus, more studies need to be devoted to exploring the meaning of technically oriented terms before applying these terms in consumer communication.

- *Communication channels*

A variety of communication channels, such as product packages, in-store displays, leaflets, manufacturers’ websites or other forms of social media, were mentioned by participants of the study and could be adopted by marketers. Participants suggested that a barcode could be put on the label so that consumers who want to learn more about the processing details could just scan and explore by themselves. Short videos (e.g., 1 min length) could be displayed in-store to explain NTP to consumers. In addition, participants showed specific interest in product tasting, which is shown to reduce the hesitance of consumers to trial purchase products processed by new technologies [26,28].

- *Communication targeted at different consumers*

Specific communication messages are recommended to reach targeted consumers of different socio-demographic backgrounds. The study suggests that different age groups may have different perceptions towards non-thermally processed F&V. Specifically, the middle-aged groups had relatively more concerns about the application of NTP, compared with the young participants. Although this cannot be inferred conclusively from a qualitative study, this finding is consistent with previous studies [19,20] which revealed that younger generations are relatively less neophobic with regard to novel technologies and food products.

Moreover, cross-cultural variation was observed in participants’ views across the six European countries. It seemed that participants from Spain, Denmark, and the Netherlands were more interested in NTP and more open to learning how it works, compared to Italy, Serbia, and Germany. This might partly depend on the food culture and the status of food safety in different countries [3,32,33,34]. The importance of various basic values such as food safety and naturalness may differ across cultures and influence consumers’ attitudes and behaviours in different ways [3]. Moreover, consumers’ confidence in their national food safety control systems varied from country to country [32,34], which might lead to the differences of confidence towards F&V processed by novel technologies. Again, however, the results are based on a qualitative study so further research, such as a large-scale consumer study, is advised to further confirm the existence of these cross-cultural differences.

### 4.2. Usage of Product Information

Besides information on NTP specifically, information associated with product quality and environmental impacts caught the most attention, including product origin, date of packaging and expiration, presence of certifications, ingredients and nutrients table (for F&V derived products), storage instructions, and recyclability of the package.

In addition to the conventional traceability information (e.g., origin, date of packaging), participants who were especially interested in sustainable development wished to know more environmental information (e.g., based on life cycle assessment) of F&V products. The literature suggests that the perceived environmental friendliness, inferable from traceability information, enhanced the perceived quality of food products [35], and our findings confirm that. Consumers prefer the simple and direct presentation of traceability information, which they can easily find and understand [36]. For instance, participants in our study preferred carbon labels that allow comparisons of carbon footprints across products, and QR codes that could present the key points of a product’s life cycle, which are consistent with findings reported by Hartikainen [37] and Tarjan [38], respectively.

However, it should be noted that sustainability labeling, at present, may still not play a significant role in the majority of consumers’ food choices. Moreover, due to social desirability bias, the extent to which consumers’ general concern about food sustainability can be turned into actual behavior is unknown [39,40]. Further studies could focus on the effects of sustainability labels on consumer perceptions and ways to promote environmentally sustainable food purchases, which could contribute to food waste reduction eventually [39].

Providing information on storage and cooking instructions was found to be potentially beneficial to F&V waste reduction through influencing consumers’ household behaviors. Our results showed that participants’ knowledge of F&V storage was not always optimal. Storage as well as cooking instructions, e.g., usage of F&V in different ripeness levels, could be inspiring to consumers.

### 4.3. Household F&V Waste Reduction and Interactive NTP

Consumers’ role within the issue of food waste is especially crucial, as recently emphasized by the new “Farm to Fork” strategy of the EU Commission [41]. Food surplus and wastage at the purchase and household stages are observed in Europe [24,42], accounting for approximately 35% of all food wasted [43]. In our study, most participants reported that due to their increased awareness of food waste and its costs on society, they have been making different efforts to reduce F&V waste and achieved a decrease in household F&V waste. Consumers expected more support from the industry and retailers to further reduce household waste. For instance, a better choice for loose F&V, and package sizes appropriate for their consumption needs [44].

It could be interesting to further explore the effects of interactive NTP (e.g., intelligent packaging with freshness-indicator) on household F&V waste reduction. Participants saw the benefits of freshness-indicator with respect to convenience, food safety, and waste reduction aspects, especially for products for which it is difficult to tell ripeness from appearance. However, it was reported that freshness indicators and other similar intelligent devices might push consumers to purchase only newly displayed foodstuffs and increase the number of unsold items [45,46]. The effects of intelligent packaging on consumers’ actual behavior could be further explored in future studies.

## 5. Conclusions

This study explored factors that influenced consumers’ perceptions and purchase willingness of non-thermally processed F&V products, in six European countries: Denmark, Italy, Serbia, Spain, Germany, and the Netherlands. Lack of basic knowledge and trust among consumers was identified as the major potential impediment to their acceptance of non-thermally processed F&V products. Consumers have difficulties in assessing relevant benefits and risks, which engenders concerns and impedes the establishment of social trust. These findings suggest that an increase in public interest in novel NTP and non-thermally processed F&V products may be a long-term process. Consumer-oriented communication and education are necessary to enhance social awareness and trust. Information that incorporates benefits for the consumers could affect consumers’ purchase willingness positively, especially when the information is concise and from trusted sources and the benefits are directly related to product quality and safety. Furthermore, consumers had a higher willingness in consuming F&V processed by more environmentally-friendly technologies which could save energy and provide benefits in terms of F&V waste reduction.

## Figures and Tables

**Table 1:** Demographic information of the participants in the six participating countries.

Country	Participants (N)	Group *	Gender Split (M/F)
Denmark	17	10 YA	5/5
		7 MA	4/3
Germany	16	8 YA	4/4
		8 MA	5/3
Italy	14	8 YA	3/5
		6 MA	2/4
Serbia	16	8 YA	4/4
		8 MA	4/4
Spain	15	7 YA	4/3
		8 MA	4/4
The Netherlands	16	8 YA	4/4
		8 MA	4/4
Total	94	49 YA	47/47
		45 MA	

* Number of participants for YA focus group and MA focus group, YA = 18–30 years old, MA = 45–60 years old.

**Table 2:** Interview protocol for the focus groups.

Discussion Themes	Subthemes
1. Participants’ preferred quality attributes of F&V	Consumption of F&V products in general; Consumers’ preferred quality attributes of F&V products;
2. Participants’ perception of non-thermal processing technologies for F&V	Consumers’ familiarity with/knowledge of NTP; Consumers’ perceptions towards non-thermally processed F&V; Expected benefits and concerns regarding NTP; Expectations regarding the communication of processing information of non-thermally processed F&V;
3. Participants’ use of processing and package information of F&V	Use of processing information and other product information of F&V during purchase and at home; Perceived importance of different information categories.
4. Participants’ household storage and waste of F&V	Consumers’ storage behavior of F&V at home in general; Reasons for discard of F&V at home; Consumers’ behavior related to reduction of F&V waste; Ideas and expectation as to how companies could contribute to their reduction of F&V waste.

**Table 3:** Relative frequency (%) of concerns and expected benefits stated by young (18–35) and middle-aged (45–60) participants in all countries.

Country	Young (18–35)		Middle Aged (45–60)	
	Concerns	Benefits	Concerns	Benefits
Denmark	44%	56%	57%	43%
Germany	92%	8%	75%	25%
Italy	94%	6%	56%	44%
Serbia	70%	30%	91%	9%
Spain	20%	80%	57%	43%
The Netherlands	56%	44%	60%	40%
Aggregated	63%	37%	66%	34%

Note: Reported figures represent the percentages of concern statements and benefit statements among all the concern and benefit statements claimed by young and middle-aged participants.

**Table 4:** Relative frequency (%) of concerns and expected benefits stated by female and male participants in all countries.

Country	Females		Males	
	Concerns	Benefits	Concerns	Benefits
Denmark	59%	41%	20%	80%
Germany	80%	20%	84%	16%
Italy	81%	19%	67%	33%
Serbia	68%	32%	100%	0%
Spain	0%	100%	47%	53%
The Netherlands	80%	20%	50%	50%
Aggregated	61%	39%	61%	39%

Note: Reported figures represent the percentages of concern statements and benefit statements among all the concern and benefit statements claimed by female and male participants.
